# Investigation of multipotent postnatal stem cells from human maxillary sinus membrane

**DOI:** 10.1038/srep11660

**Published:** 2015-06-29

**Authors:** JunBing Guo, JunQuan Weng, Qiong Rong, Xing Zhang, ShuangXi Zhu, DaiYing Huang, Xiang Li, Song Ling Chen

**Affiliations:** 1 Department of Oral and Maxillofacial Surgery, the First Affiliated Hospital, Sun Yat-sen University, Guangzhou, PR China; 2 Guangdong Key Laboratory of Stomatology, Sun Yat-sen University, Guangzhou, PR China; 3 Department of Prosthetic Dentistry, the First People’s Hospital of Yunnan, Kunming, PR China; 4 Department of Stomatology, Guangdong Provincial Hospital of Traditional Chinese Medicine, Guangzhou, PR China

## Abstract

Maxillary sinus membrane (MSM) elevation is a common surgical technique for increasing bone height in the posterior maxilla prior to dental implant placement. However, the biological nature of bone regeneration in MSM remains largely unidentified. In this study, MSM tissue was obtained from 16 individuals during orthognathic surgery and used to isolate MSM stem cells (MSMSCs) by single-colony selection and STRO-1 cell sorting. The cell characteristics in terms of colony-forming ability, cell surface antigens, multi-differentiation potential and *in vivo* implantation were all evaluated. It was found that MSMSCs were of mesenchymal origin and positive for mesenchymal stem cell (MSC) markers such as STRO-1, CD146, CD29 and CD44; furthermore, under defined culture conditions, MSMSCs were able to form mineral deposits and differentiate into adipocytes and chondrocytes. When transplanted into immunocompromised rodents, MSMSCs showed the capacity to generate bone-like tissue and, importantly, maintain their MSC characteristics after *in vivo* implantation. These findings provide cellular and molecular evidence that MSM contains stem cells that show functional potential in bone regeneration for dental implant.

Inadequate alveolar bone is a common limitation for inserting dental implants in the posterior maxilla^1^,^2^. Clinical and animal studies have shown that successful bone augmentation can be achieved by simply elevating the maxillary sinus membrane (MSM), with or without any bone grafting^3^,^4^,^5^. On the other hand, case reports have described spontaneous bone formation on the maxillary sinus floor following cyst and tooth removal^6^,^7^. Herein, maxillary sinus membrane elevation combined with different osteoconductive bone grafts has become a common surgical technique for increasing bone height^8^,^9^ (Fig. 1B). However, the cellular basis for this putative activity is unclear.

Mesenchymal stem cells, with the capacity of self-renewal and multi-lineage differentiation, play a crucial role in tissue engineering and regenerative medicine^10^. Stem-cell-based tissue engineering has been performed in the animal model for many types, such as articular cartilage, bone, muscle and adipose tissues^10^. Studies, including direct cell-pellet implantation^11^,^12^ and tissue regeneration in combination with biocompatible scaffolds^13^, have enabled us to contemplate new and promising strategy for tissue engineering and cell therapy. STRO-1, one of the most well-known mesenchymal stem cell markers, has gained increasing interest in stem cell sorting over the past decade^14^,^15^,^16^. For instance, STRO-1 has been utilized for the selection of periodontal ligament stem cells (PDLSCs)^14^, dental pulp stem cells (DPSCs)^15^, and bone marrow stromal stem cells (BMSSCs)^16^.

As shown in Fig. 1A, there are three layers in the maxillary sinus: 1. Pseudostratified columnar epithelium (respiratory type epithelium), 2. A highly vascular lamina propria, and 3. Periosteum. Seromucinous glands are also present in the lamina propria and empty directly into the maxillary sinus via excretory ducts. Recent findings show that osteoprogenitor cells derived from MSM have osteogenic potential, including the capacity to form bone tissue *in vivo*^17^,^18^,^19^. On the other hand, Gruber *et al*.^20^ showed that cells derived from maxillary sinus membrane express STRO-1, and respond to BMP-6 and BMP-7. From these results, we assumed that maxillary sinus membrane might contain progenitor cells that maintain tissue homoeostasis and allow for the regeneration of bone tissue.

Herein, we report the isolation and characterization of a unique stem-cell population from MSM tissue. The significance of this study is that it provides evidence indicating that the MSM is similar in some ways to bone marrow, containing multipotent stem cells that could be used for bone regeneration in the atrophic posterior maxilla.

## Results

### Isolation of clonogenic populations of MSMSCs

**Figure 2:**
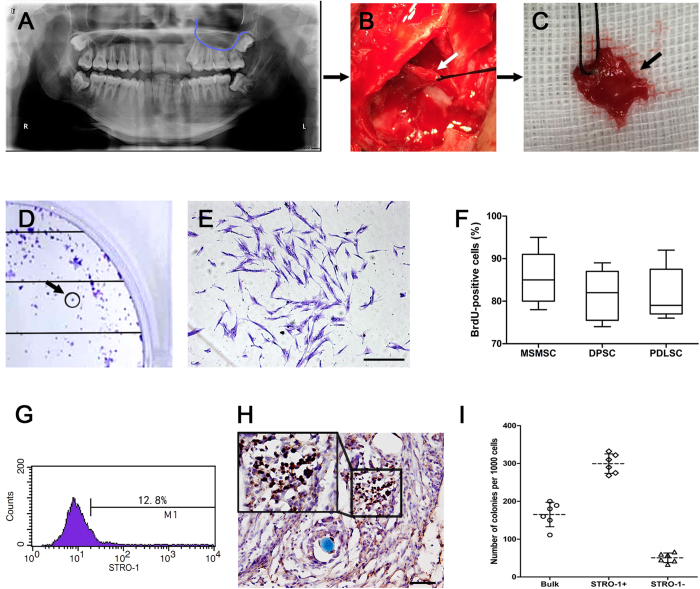
Isolation of adult human MSMSCs. (**A**–**C**) Schematic of the procedure for obtaining normal human MSM tissue during orthognathic surgery (**D**) Single colonies formed after MSMSCs were plated at low density. (**E**) A single colony-forming unit of MSMSCs when observed using a stereomicroscope. (**F**) Bromodeoxyuridine (BrdU) labelling efficiency of MSMSCs, DPSCs and PDLSCs were assessed by BrdU incorporation for 24 h. The number of BrdU-positive cells was expressed as a percentage of total number of cells counted from six replicate cultures as shown in boxplot. MSMSCs showed a higher uptake rate than did DPSCs or PDLSCs, but there was no significant statistical difference (p>0.05). Horizontal lines are median values. Bars show maximum and minimum values. (**G**) Flow cytometric analysis of STRO-1, an early mesenchymal progenitor marker, in cultured MSMSCs. (**H**) MSM tissue was positive for STRO-1 antibody with immunohistochemical staining. (**I**) Clonogenic assays were subsequently done with unfractionated (bulk), STRO-1 negative (STRO-1^–^) and STRO^−^1 positive (STRO-1^+^) cell fractions. Data obtained from six individual MSM samples are shown in scatter dot plot. Horizontal lines are median values. Scar bars represent 100 μm.

To identify putative stem cells, single-cell suspensions were generated from human MSM (Fig. 2A–C). The ability of human MSM-derived cells to form adherent clonogenic cell clusters of fibroblast-like cells, similar to those recorded for different mesenchymal stem-cell populations, was demonstrated by the formation of approximately 178 single colonies (Fig. 2D,E), generated from 1 × 10^3^ single cells cultured at low density (Fig. 2I). On the other hand, ex vivo-expanded MSMCs expressed the cell surface molecule STRO-1, one early mesenchymal stem cell marker, also presented on PDLSCs^14^, DPSCs^15^ and BMSSCs^16^ (Fig. 2G). STRO-1-positive cells were also shown to be located in the MSM tissue by immunohistochemical staining (Fig. 2H). This STRO-1-positive and colony-forming cell population, which we preliminarily termed maxillary sinus membrane stem cells (MSMSCs), had a high uptake rate of bromodeoxyuridine, similar to the rate observed with DPSCs and PDLSCs (Fig. 2F). When anti-STRO-1 antibody was used to isolate MSMSCs, most colony-forming cells were found to be contained within the STRO-1-positive cell population, confirming STRO-1 as an early progenitor marker for MSMSCs (Fig. 2I).

### Characterization of the immunophenotype of MSMSCs *in vitro*

Immunocytochemical studies were performed to characterize the progeny of MSMSC clonogenic populations, and then compared with BMSSCs, which are known as precursors of osteoblasts, by using a large panel of antibodies specific to known antigens associated with different phenotypes including haematopoietic system, smooth muscle, nerve, cartilage, fat, endothelium, fibroblasts, and bone. Typical immunoreactivity profiles for both cell populations are shown in Table 1. Both populations displayed a negative profile of protein expression for the haematopoietic markers CD14 (monocyte/macrophage), CD34 (haematopoietic/endothelium), and CD45 (leucocyte) and other markers such as MyoD (smooth muscle), Nestin (nerve), Collagen-II (cartilage), and PPARγ-2 (fat). In general, MSMSCs and BMSSCs exhibited a positive expression pattern for a variety of markers associated with endothelium (VCAM-1 and CD146), fibroblasts (Collagen-III and bFGF), and bone (ALP, Collagen-I, OCN and ON). The epithelial-derived stem cell marker CK-19 was present at low levels in MSMSCs, but absent in BMSSCs. Additionally, MSMSCs could not express BSP (osteoblast marker), whereas positive immunostaining could be detected in BMSSCs. Representative immunoreactivity patterns for MSMSCs are shown (Supplementary material Fig. S1). Many of the markers were not uniformly expressed but were found in subsets of cells, as has been shown for the BMSSC population.

### Osteogenic potential

**Figure 3:**
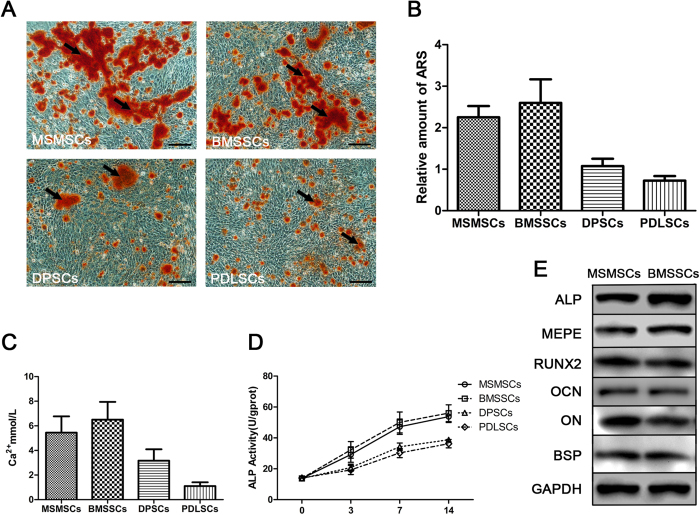
Osteogenic differentiation of MSMSCs; BMSSCs, DPSCs and PDLSCs served as matched controls. (**A**) Representative images of mineralized nodules formed after 4 weeks of osteogenic induction (stained with Alizarin Red). (**B**) The total areas of mineralized nodules in different groups were quantified using cetylpyridinium chloride. (**C**) Calcium concentration in different groups was measured. (**D**) ALP activity at various culture times. (**E**) Western blot analysis of osteoblastic markers after 4 weeks of osteogenic induction, including ALP, MEPE, RUNX2, OCN, ON and BSP, GAPDH was used to assess the amount of protein loaded per sample. Scar bars represent 100 μm.

To investigate the potential of MSMSCs to undergo osteoblastic differentiation, established secondary MSMSCs were treated with osteogenic induction medium to induce mineralization *in vitro*. Small round alizarin red-positive nodules formed in the MSMSC cultures after 4 weeks of induction, indicating calcium accumulation *in vitro* (Fig. 3A). Although the mineralized nodules appeared to be slightly low in number for MSMSCs than BMSSCs, the difference was not statistically significant (Fig. 3B; p = 0.1584). However, compared with DPSCs and PDLSCs, MSMSCs formed more nodules, which correlated with higher concentrations of calcium in the extracellular matrix (Fig. 3C, p = 0.0036 or p = 0.0003). Western blot analysis (Fig. 3E) showed that cultured MSMSCs expressed an array of osteoblastic markers, including ALP, MEPE, RUNX2, OCN, ON, and BSP. ALP activity is believed to be an important indicator for osteoblast differentiation^21^. As shown in Fig. 3D, ALP activity in every group increased with culture time; the results indicate that there was no significant difference between the MSMSC and BMSSC groups at each time point. However, at days 3, 7 and 14, the ALP levels in the MSMSC group were significantly higher than those in the DPSC and PDLSC groups, implying that cell differentiation may be more active in MSMSC cells.

### Adipogenic potential

**Figure 4:**
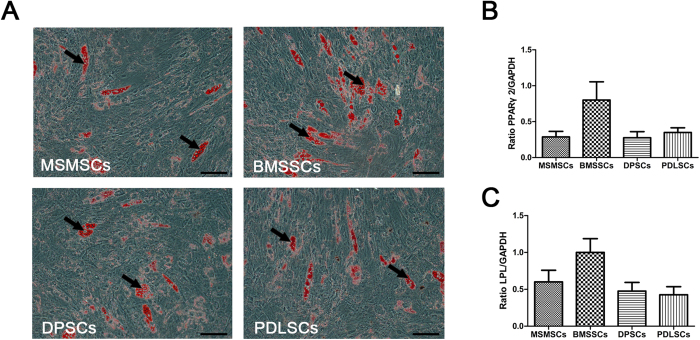
Adipogenic differentiation of MSMSCs; BMSSCs, DPSCs and PDLSCs served as matched controls. (**A**) Representative images of intracellular lipid vacuoles that appeared in all tested cells, as confirmed by Oil Red O staining. (**B**) and (**C**) The expression of adipogenic markers PPARγ-2 and LPL detected by RT-PCR. Scar bars represent 100 μm.

We assessed whether MSMSCs, like BMSSCs, DPSCs, and PDLSCs had the potential to differentiate into other cell lineages such as adipocytes. After 3 weeks of culture with an adipogenic induction medium, MSMSCs developed into oil red O-positive lipid-laden fat cells (Fig. 4A). This development correlated with an upregulation in the expression of two adipocyte specific transcripts, PPARγ-2 and lipoprotein lipase, as detected by qPCR (Fig. 4B,C).

### Chondrogenic potential

**Figure 5:**
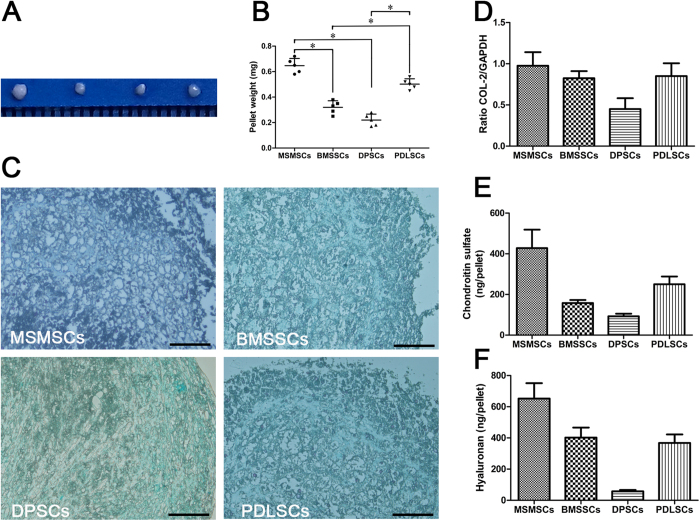
Chondrogenic differentiation of MSMSCs; BMSSCs, DPSCs and PDLSCs served as matched controls. (**A**) Representative cell pellets (1 mm scale). (**B**) Wet weight of cell pellets. Data obtained from five individual cell pellets are shown in scatter dot plot. Horizontal lines are median values. (**C**)Histology of pellets stained with toluidine blue. (**D**) The expression of a chondrogenic marker COL-2 detected by RT-PCR. (**E**) and (**F**) Amount of chondroitin sulphate and hyaluronan in pellet cultures. Scar bars represent 100 μm.

*In vitro* chondrogenesis, in a pellet culture system, was performed to evaluate the chondrogenesis potential of MSMSCs, BMSSCs, DPSCs, and PDLSCs. During chondrogenesis, the pellets increased in size because of the production of extracellular matrix^22^. The pellets derived from MSMSCs and PDLSCs were larger and heavier than those derived from BMSSCs (Fig. 5A,B p = 0.0084 and p = 0.0105), and DPSCs (Fig. 5A,B p = 0.0023 or p = 0.0058), indicating their superiority in chondrogenesis. Histologically, each cell pellet exhibited a cartilage matrix that was stained with toluidine blue (Fig. 5C). The expression of COL-2 mRNA in DPSCs appeared lower than that in other cell types (Fig. 5D). The syntheses of chondroitin sulfate and hyaluronan were the highest in MSMSCs and the lowest in DPSCs (Fig. 5E,F).

### *In vivo* transplantation

**Figure 6:**
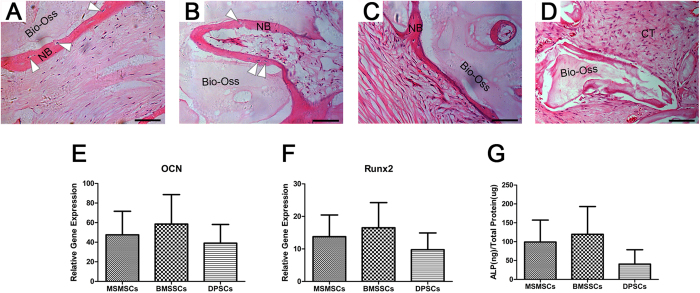
*In vivo* transplantation of MSMSCs, BMSSCs and DPSCs. (**A**) In the MSMSC transplants, HE staining shows that new bone-like structure (NB) with osteocyte-like cells (triangles) embedded within the calcified matrix was formed on the surface of Bio-Oss carrier. (**B**) and (**C**) BMSSC and DPSC transplants were used as controls to show the formation of new bone-like structure (NB) containing osteocyte-like cells (triangles). (**D**) Of 15 selected strains of single-colony derived MSMSC, only nine (60%) showed a capacity to form a bone-like tissue. The other six strains did not generate mineralized tissues *in vivo*. (**E**) and (**F**) Relative expressions of osteogenic differentiation markers Runx2 and OCN in the MSMSCs transplants. The gene expression level of Bio-Oss transplants was set as the control (normalized to 1). (**G**) Relative quantity of active ALP protein to the total proteins in the transplants. Scar bars represent 100 μm.

To validate the capacity of MSMSCs to differentiate into functional osteoblast-like cells, MSMSCs were transplanted in conjunction with Bio-Oss scaffolds into immunocompromised mice. A bone-like structure was regenerated, in which a thin layer of bone-like tissue, containing elements of bone and osteocyte-like cells, formed on the surface of the carrier, along with condensed collagen fibres with sparse cells that resembled periosteum-like structures (Fig. 6A). Different degrees of new bone formation were also observed in BMSSC and DPSC transplant (Fig, 6B,C). In contrast, no bone formation or osteoblast was observed in the control Bio-Oss (Fig. 6D). Of 15 single-colony-derived MSMSC clones transplanted into immunocompromised mice, 9(60%) showed a capacity to form bone-like tissues, equivalent to multicolony-derived MSMSCs. The remaining six clones did not form bone-like tissues.

Moreover, the expressions of osteoblast-differentiation-related genes (Runx2 and OCN) appeared to be higher in BMSSCs, but the difference was not statistically significant (Fig. 6E,F). However, ALP analysis revealed that the amount of ALP was significantly greater in MSMSCs than in DPSCs (Fig. 6G, p = 0.0089).

### Characterization of re-MSMCs

**Figure 7:**
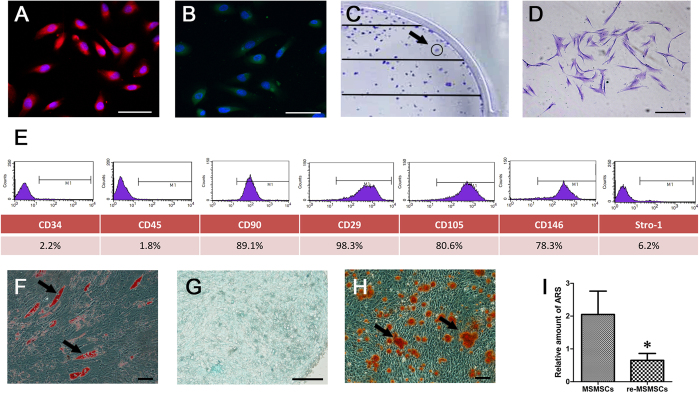
Characterization of cells obtained from *in vivo*-regenerated MSM-like tissues (re-MSMCs). (**A**) and (**B**) Immunofluorescence staining showed that re-MSMCs positively interacted with the anti-human mitochondria and vimentin antibodies. (**C**) Single colonies formed after re-MSMCs were plated at low density. (**D**) Representative images of a single colony-forming unit of re-MSMCs. (**E**) Cell surface markers of re-MSMCs; representative figures of cytometric flow tests and percentage of positive expression. (**F**–**H**) Representative images of adipogenic, chondrogenic and osteogenic differentiation of re-MSMCs. (**I**) The total areas of mineralized nodules were quantified by densitometry. *P < 0.05 indicates significant differences between two matched groups. Scar bars represent 100 μm.

Re-isolated MSMCs from *in vivo*-regenerated tissues were positively interacted with the anti-human mitochondria antibody, indicating that the re-MSMCs obtained in the present study were of human origin (Fig. 7A). Moreover, the majority of the re-isolated cells positively interacted with the vimentin antibody and retained their colony-forming ability, indicating their mesenchymal origin (Fig. 7B–D). Re-MSMCs also positively expressed MSC markers, such as STRO-1, CD146, CD29, CD105 and CD90, while negatively expressing haematopoietic cell markers, such as CD34 and CD45, but their expression of STRO-1 was lower than that of MSMSCs (Fig. 7E, P = 0.0217). Moreover, re-MSMCs were still able to form mineral deposits and differentiate into adipocytes (Fig.7F) and chondrocytes (Fig.7G), although quantitative analysis showed that the osteo-differentiation capabilities of the re-MSMCs were significantly reduced compared to those of the MSMSCs (Fig. 7H,I, P = 0.0156).

## Discussion

Recent advances in stem cell biology, together with bio-inspired materials design, have offered new insights into the understanding of bone forming and have expanded opportunities for the therapeutic application of tissue engineering. Clinical observations of bone formation in sinus lifting procedures with or without adjunctive bone grafts have been reported^8^,^9^. However, the precise cell properties and the underlying mechanisms that determine the lineages-pecific differentiation of human MSM-derived cells are either unexplored or partially explored. Our findings show that human MSM contains a population of multipotent postnatal stem cells that can be isolated and expanded *in vitro*, providing a unique reservoir of stem cells.

Although their localization behaviours in situ are quite different, MSMSCs and BMSSCs share many features in common. Potent regulators of bone formation of BMSSCs such as transforming growth factor-b, bone morphogenic protein (BMP)-2, and BMP-6 have also been implicated as promoters of bone regeneration in MSM cells^9^,^19^,^20^. Other growth factors believed to regulate the proliferation and differentiation of MSM cells include simvastatin, platelet-derived growth factor, and insulinlike growth factor I, all of which affect osteoblastic cells as well^23^,^24^,^25^. On the other hand, BMSSCs and DPSCs have been characterized as STRO-1positive progenitors derived from a perivascular niche within the bone marrow and dental pulp microenvironments^19^,^20^. Similarly, the deeper portion of the maxillary sinus membrane is a highly vascularized connective tissue, and STRO-1-positive cells have also been shown to be located in MSM tissue. Moreover, it is of interest to note that MSMSCs express smooth muscle and endothelial markers, similarly to BMSSCs. Based on studies in developmental biology, it has been suggested that these stem cells may arise from developing blood vessels^26^,^27^,^28^. Further characterization of MSMSCs by using current molecular technology will hopefully provide novel markers that will be useful in their identification in situ and their isolation and purification ex vivo.

It is important to consider that, although different types of tissue-derived MSCs share several common multidifferentiation potentials, differences have been noted between different stem cell types. The present study demonstrates that human MSMSCs do share a similar pattern of protein expression with BMSSCs *in vitro*. However, several subsets of cells expressing markers of smooth muscle (a-SM actin), and endothelial cells (CD146 and VCAM-1) were represented in both MSMSCs and BMSSCs. This result is consistent with the findings in BMSSCs and DPSCs^26^,^29^,^30^. One possible explanation for this heterogeneous immunophenotype in MSMSCs is that the colony-forming STRO-1^+^ cells within MSM tissue are derived from different pluri-potent stem cell pool.

Notably, the ability of stem cells to maintain their self-renewal and multi-lineage differentiation potential *in vitro* and *in vivo* is of great importance in ensuring effective tissue regeneration^23^,^30^. Evidence suggests that a number of factors, including the ability of MSCs to undergo self-renewal, determine the cell capacity for long-term survival and participation in tissue formation following *in vivo* transplantation^23^,^31^. Previous study have described that MSM contains osteoprogenitor cells and capable of generating bone-like tissue^19^. However, in this previous study, the stem cell properties, in terms of their colony-forming ability, cell surface antigens and multi-differentiation potentials, were not contrastively evaluated before and after implantation, and cells used in this previous study are heterogeneous unsorted. Unselected MSCs contain multiple cell types including nonmesenchymal stem cell types, such as epithelial cells, inflammatory cells, hematopoietic stem cells, and endothelial cells, which may affect their characterization and application^32^. Therefore, our study used colony selection and STRO-1 marker to isolate MSMSCs from MSM tissue, and showed that MSMSCs represent a population of multipotent stem cells, as demonstrated by their capacity to give rise to osteogenic, adipogenic and chondrogenic lineages *in vitro* and bone-like tissue *in vivo*.

The Bio-Oss used in our transplantation system may be “osteo”-conductive for MSMSCs, BMSSCs and DPSCs. When coimplanted with Bio-Oss scaffolds, they are all able to form bone like material *in-vivo*. However, of 15 selected strains of single-colony-derived MSMSC, only 9(60%) showed a capacity to form bone-like tissue *in vivo*, several alternative explanations are possible. For example, because of the heterogeneity of STRO-1 in sorting mesenchymal stem cells^26^, it is possible that the MSMSCs used in our experiments represent a heterogeneous stem-cell-enriched population that contains some early progenitor cells, with restricted differentiation potential which leads to unreliable bone formation *in vivo*. Another interpretation is that the clonal cell isolation, 15 to 20 population doublings, and *in vitro* culture conditions may have caused some loss of multilineage potential that was originally present in these cells. Therefore, more work is clearly needed to find out specific markers that identify different developmental stages during osteogenesis, particularly for primitive subpopulations and to assess the full developmental potential of different MSMSC clones, in analogy to the proposed marrow stromal hierarchy of cellular differentiation^33^, in which only a subset of BMSSC colonies have the potential to develop into multiple cell lineages *in vitro*, or the capacity to form new bone and marrow elements *in vivo*^22^.

There’s evidence to suggest that MSC could maintain self-renewal capability and multipotent potential and even undergo a long-term period of *in vivo* transplantation^34^,^35^,^36^. For instance, stromal-like cells were reestablished in culture from primary DPSC transplants and were able to generate dentin pulp-like tissues when retransplanted into immunocompromised mice^34^. In a recent study, PDLSCs were demonstrated to survive after 8 weeks of posttransplantation into immunodeficient mice. These cells exhibited a multipotential capacity and displayed a capacity to form fibrous ligament structures and mineralized tissues comparable to primary PDLSCs^35^. In the present study, we found that re-MSMCs derived from regenerated tissue in immunocompromised mice were still of human and mesenchymal origin, and positive for MSC markers such as STRO-1, CD146, CD29, CD90 and CD105 and, to some extent, re-MSMCs maintained their colony forming abilities. On the other hand, re-MSMCs were able to form mineral deposits and differentiate into adipocytes and chondrocytes *in vitro*. These findings suggesting that these cells may belong to a population of more primitive reserve cells that are responsible for the maintenance of stem cell properties or are indeed daughter cells that are generated by the originally transplanted stem cells. However, there is currently no consensus regarding the effect of *in vivo* transplantation on this specific MSC function, and the underlying mechanisms that determine the perseverance of lineage-specific differentiation of MSCs are still unclear.

In conclusion, the data presented here demonstrate that postnatal MSMSCs are clonogenic, highly proliferative cells that have the ability to differentiate into specialized lineages and are capable of regenerating tissue, properties that effectively define them as stem cells. Importantly, our results show that human MSMSCs participate in the process of bone regeneration in immunocompromised mice, and these cells have the potential to retain their stem cell-like properties after long-term *in vivo* transplantation. However, the biological mechanism of bone regeneration is unclear. In future studies, molecules and pathways to optimize the bone regeneration capacity of these cells should be investigated after maxillary sinus lifting.

## Methods

### Ethics statement

All protocols and the informed consent form for MSM isolation were approved by the Sun Yat-Sen University Joint Institutional Review Board and performed in accordance with the guidelines of the Medical Ethics Committee of Sun Yat-Sen University. The specimen donors were provided the IRB-approved formal consent form describing sufficient information for one to make an informed decision about his/ her participation in this study. The formal consent forms were signed by the subjects before specimen collection.

### Antibodies

Antibodies against human antigens CD14, CD34, CD44, CD45, CD146, CD90, CD29, CD105 and CD106 were purchased from BD Biosciences (San Jose, CA, USA). An antibody against human vimentin was obtained from GeneTex (Taiwan). An antibody against mitochondria was purchased from Millipore (Billerica, MA, USA). Antibodies against STRO-1, RUNX2, COL-I, COL-II, COL-III, ALP, BSP and PPARγ-2 were obtained from Abcam (Cambridge, CB4 0FW, UK). Antibodies against OCN, ON, OPN and GAPDH were purchased from Cell Signaling Technology (Danvers, MA, USA). Antibodies against MyoD, Nestin, bFGF, CK-19 and MEPE were purchased from Santa Cruz Biotechnology Inc. (Santa Cruz, CA, USA).

### Samples and cell culture

Normal human MSM samples were obtained with informed consent from 16 individuals aged 18–29 years during orthognathic surgery in the Department of Oral and Maxillofacial Surgery of the First Affiliated Hospital, Sun Yat-sen University. Smokers or patients with syndromatic diseases were excluded. The tissues were minced and then digested in a solution of 3 mg/mL collagenase type I (Sigma, Lakewood, N.J., USA) and 4 mg/mL dispase (Roche, Mannheim, Germany) for 1 h at 37 °C. MSM samples were pooled and single-cell suspensions were obtained by passing the cells through a 70 μm strainer (Falcon, BD Labware, Franklin Lakes, NJ, USA). Single-cell suspensions (1 × 10^4^ cells) were seeded into 75-cm^2^ culture dishes (Costar, Cambridge, MA, USA) with alpha modification of Eagle’s medium (GIBCO BRL, Grand Island, NY, USA) supplemented with 20% fetal calf serum (Hyclone, Logan, UT, USA), 100 μmol/L ascorbic acid 2-phosphate, 2 μmol/L glutamine, 100 U/mL penicillin, and 100 μg/mL streptomycin (Biofluids, Rockville, MD, USA); the suspensions were then incubated at 37 °C in 5% carbon dioxide.

BMSSCs were obtained from ScienCell Research Laboratory (San Diego, CA, USA). DPSCs and PDLSCs were isolated and cultured as previously described^34^,^35^. Briefly, normal human impacted third molars (n = 22) extracted for orthodontic or orthognathic reasons were collected from healthy donors aged 18 to 29 years. Periodontal ligament was gently separated from the surface of the root, and then the teeth were cut and the pulp tissue was gently separated from the crown and root and minced into small pieces. The small fragments were digested in a solution of 3 mg/mL collagenase type I (Sigma, Lakewood, N.J., USA) and 4 mg/mL dispase (Roche, Mannheim, Germany) for 1 h at 37 °C. In some experiments, MSMSCs, DPSCs and PDLSCs were obtained from the same donor or donors. BMSSCs were obtained from ScienCell Research Laboratory (San Diego, CA, USA). All primary cells used in this study were submitted to 2–4 passages. For each experiment, the same passage of MSMSCs, BMSSCs, DPSCs, and PDLSCs was used.

### Single-cell cloning and proliferation assay

To assess colony-forming efficiency, 1 × 10^3^ cells at Passage 1 were cultured for 7 days in 60-cm^2^ dishes, and then stained with 0.1% crystal violet. Aggregates of ≥50 cells were scored as colonies. The proliferation rate of sub-confluent cultures (first passage) of stem cells was assessed by bromodeoxyuridine incorporation for 24 h, with a Zymed BrdU staining kit (Zymed Laboratories, San Francisco, CA, USA).

### Immunomagnetic isolation

To obtain STRO-1^+^ stem cells, each cell populations at the second passage were indirectly sorted using immunomagnetic beads (Dynabeads; Dynal Biotech, Oslo, Norway) according to the manufacturer’s instructions. Briefly, single-cell suspensions were incubated with STRO-1 supernatant (mouse anti-human BMSSCs, IgM) for 1 h on ice. The cells were then washed with phosphate buffered saline containing 5% bovine serum albumin, and resuspended with rat anti-mouse IgM-conjugated Dynal beads at four beads per cell for 60 min on a rotary mixer at 4 °C. Bead-positive cells were segregated with a magnetic particle separator and subsequently seeded into 75-cm^2^ culture flasks (Costar, Cambridge, MA, USA) at 37 °C in 5% CO_2_.

### Flow cytometry

Approximately 1 × 10^6^ of *in-vitro* expanded MSMSCs were suspended in 500 μl PBS containing 20 ng/ml fluorescein isothiocyanate (FITC)-coupled antibodies against CD34, CD45, CD146, CD90, CD29, CD105 Stro-1 and, as an isotype control, FITC-coupled nonspecific IgG (Becton Dickinson, Franklin Lakes, N.J., USA). After incubation for 30 min at 4 °C, the cells were washed with PBS and resuspended in 1 ml PBS for analysis using a FACSCalibur instrument.

### Differentiation assays

For osteogenic, adipogenic and chondrogenic differentiation, commercially differentiation media kits (StemPro Adipogenesis, Chondrogenesis and Osteogenesis Kits; Invitrogen) were used in accordance with the manufacturer instructions. Briefly, cells were seeded at densities of 1 × 10^4^ (osteocyte) and 3.2 × 10^4^ (adipocyte) cells/cm^2^; for chondrocyte differentiation, cells were seeded at 5 × 10^5^ cells in a 15 ml polypropylene tube (Falcon, Bedford, Mass., USA). For osteocyte differentiation, cells were fixed in 4% paraformaldehyde, stained with fresh 2% alizarin red solution (pH 4.2) for 5 minutes, and then de-stained in 10 mmol L^−1^ sodium phosphate containing 10% cetylpyridinium chloride (Sigma Diagnostics, St Louis, MO, USA). The amount of alizarin red stain was quantified by measuring the absorbance of the solution at 562 nm. The calcium concentration was measured with a Sigma calcium kit 587-A (Sigma Diagnostics, St Louis, MO, USA). For adipocyte differentiation, cells were fixed in 4% paraformaldehyde for 15 min, and stained with fresh Oil Red-O solution for 15 minutes. For chondrocyte differentiation, the pellets were embedded in paraffin, cut into 5-μm-thick sections, and stained with 1% toluidine blue. The concentrations of chondroitin sulfate and hyaluronan were quantified as reported previously^15^.

### Alkaline phosphatase activity analysis

Cells were seeded into 96-well plates (Costar, Cambridge, MA, USA) at a density of 2 × 10^4^ cells/well. After 3-, 7- and 14- days of culture in calcification medium, ALP activity was detected using an ALP assay kit (JianCheng Co., Nanjing, China) according to the manufacturer’s instructions. The amount of ALP in the cells was normalized against the total protein content.

### Western blotting

The primary antibodies used for western blotting were as described above including ALP (1:500), MEPE (1:500), RUNX2 (1:500), OCN (1:500), ON (1:500), BSP (1:200), GAPDH (1:1000). Western blot analyses were carried out as previously reported^9^.

### Real-time quantitative PCR

Total RNA isolation, first-strand cDNA synthesis, and PCR processes were performed as described previously^24^. The primer sequences were as follows: PPARγ-2 forward 5’-CTCCTATTGACCCAGAAAGC-3’, reverse 5’-GTAGAGCTGAGTCTTCT-CAG-3’; LPL forward 5’-ATGGAGAGCAAAGCCCTGCTC-3’, reverse 5’-GTTAGGTCCAGCT-GGATCGAG-3’; RUNX2 forward 5’-CACTGGCGCTGCAACAAGA-3’, reverse 5’-CATTCCGG-AGCTCAGCAGAATAA-3’; OCN forward 5’-CATGAGAGCCCTCACA-3’, reverse 5’-AGAGCG-ACACCCTAGAC-3’; COL-2 forward 5’-CCAGTTGGGAGTAATGCAAGGA-3’, reverse 5’-ACACCAGGTTCACCAGGTTCA-3’; GAPDH forward 5’-AGCCGCATCTTCTTTTGCGT C-3’, reverse 5’-TCATATTTGGCAGGTTTTTCT-3’;

### Immunofluorescence assays

The cells were fixed in 4% paraformaldehyde and then blocked and incubated with primary antibodies for 1 h. The samples were subsequently incubated with fluorescence-tagged secondary antibodies (Jackson ImmunoResearch, West Grove, PA, USA), for 45 min. The stained samples were mounted with a medium containing DAPI (Vector Laboratories Inc., Burlingame, CA, USA) and imaged using an epifluorescence microscope (Leica, Wezlar, Germany).

### Transplantation

The animal study was approved by the Ethical Committee on Animal Research of the Sun Yat-Sen University. All experimental procedures were performed according to national guidelines regarding the care and use of laboratory animals. Specific-pathogen-free (SPF) male immunocompromised mice (n = 10, 5-week-old, BALB/c-nu) were purchased from the Experimental Animal Department of the Chinese Academy of Sciences, and were maintained in SPF conditions in the whole process of experiment. Approximately 4 × 10^6^ *in vitro*- expanded MSMSCs were combined with 40 mg of deproteinized bovine bone (Bio-Oss; Geistlich, Wolhusen, Switzerland) and then transplanted into dorsal subcutaneous pockets (maximum four for each mouse), as described in previous studies^26^. Briefly, the mice were anesthetized, and the experimental materials were then transplanted into subcutaneous pockets on the back of each mouse. Bio-Oss deproteinized bovine xenograft particles placed alone constituted a negative control. All transplants were retrieved after 8 weeks and divided into two or three fragments for further analysis. For histology analysis, the transplant fragments (n = 12) were fixed with 10% buffered formalin and then decalcified with 10% edetic acid (pH 8.0), for 2 weeks. The decalcified transplants were embedded in paraffin, sectioned to a thickness of 3 μm, and stained with haematoxylin and eosin. An ALP assay kit was used to measure the ALP levels in the transplants according to the manufacturer’s instructions. The relative expressions of the genes encoding Runx2, OCN, and GAPDH were evaluated by quantitative PCR.

### Characterization of re-MSMCs

To obtain cells from *in vivo*-regenerated tissues, the newly formed tissues inside the transplants were minced and digested in a solution of 3 mg/mL collagenase type I and 4 mg/mL dispase (Sigma, St Louis, MO, USA) for 1 h at 37 °C. The procedure for cell re-isolation was similar to that for MSM cell isolation. Immunofluorescence assays were used to determine the origin of these cells; flow cytometry was performed to analyse the surface antigen expression of re-MSMCs, and multi-differentiation potentials were evaluated as described above.

### Statistical analysis

Each experiment was performed in triplicate and repeated at least three times. Experimental groups were compared using one-way analysis of variance (ANOVA) or repeated measures ANOVA with SPSS software (SPSS, Chicago, IL, USA). Values are expressed as means ± SD. All P-values are two-tailed, and P < 0.05 was considered statistically significant.

## Additional Information

**How to cite this article**: Guo, J. B. *et al*. Investigation of multipotent postnatal stem cells from human maxillary sinus membrane. *Sci. Rep*. **5**, 11660; doi: 10.1038/srep11660 (2015).

## Supplementary Material

Supplementary Information

## Figures and Tables

**Figure 1:**
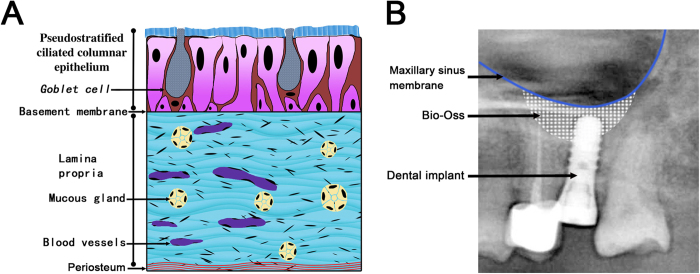
Schematic of human Maxillary sinus membrane (MSM) and radiograph of MSM elevation. (**A**) Schematic representation showing the pseudostratified epithelium, lamina propria, and periosteum components of the MSM. (**B**) Postoperative radiograph showing significant elevation of the MSM and dental implant placement with Bio-Oss.

**Table 1:** Immunohistochemical analysis of human MSMSCs and BMSSCs *in vitro*.

Marker	MSSMSCs	BMSSCs
**CD14**	**−**	**−**
**CD34**	**−**	**−**
**CD44**	**++**	**++**
**CD45**	**−**	**−**
**Integrin β1**	**++/+**	**++**
**MyoD**	**−**	**−**
**a-SM actin**	+/**−**	**++/+/−**
**Nestin**	**−**	**−**
**VCAM-1**	**++/+**	**++**
**CD146**	**++/+**	**++/+/−**
**Collagen-III**	**++/+**	**++/+**
**bFGF**	**++/+**	**++/+**
**CK-19**	**+/−**	**−**
**ALP**	**++/+**	**++/+**
**Collagen-I**	**+**	**++/+**
**OCN**	**++/+**	**+**
**ON**	**++**	**++/+**
**BSP**	**−**	**+/−**
**Collagen-II**	**−**	**−**
**PPARγ-2**	**−**	**−**

++, strong staining; +, weak staining; -, negative; MyoD, myocyte origin; VCAM-1, vascular cell adhesion molecule 1; bFGF, basic fibroblast growth factor; CK-19, cytokeratin 19; ALP, alkaline phosphatase; OCN, osteocalcin; ON, Osteonectin; BSP, bone sialoprotein; PPARγ-2, peroxisomal proliferator activated receptor gamma 2;.
