# Supplementary material for: Investigation of multipotent postnatal stem cells from human maxillary sinus membrane
Source: Sci Rep. 2015 Jun 29;5:11660. doi: 10.1038/srep11660 (PMC4484356; doi:10.1038/srep11660)

Investigation of multipotent postnatal stem cells from human maxillary sinus membrane

JunBing Guo[a,b,1], JunQuan Weng[a,b,1], Qiong Rong[c], Xing Zhang[d], ShuangXi Zhu[a,b], DaiYing Huang[a,b], Xiang Li[a,b], SongLing Chen[a,b,*]

Supplementary material Fig. S1 Immunophenotype of cultured MSMSCs.

Representative staining patterns are shown for: (A) CD44; (B) Integrin β1; (C) a-SM actin; (D) VCAM-1; (E) CD146; (F) Collagen-III; (G) bFGF; (H) CK-19; (I) ALP; (J) Collagen-I; (K) OCN; (L) ON.

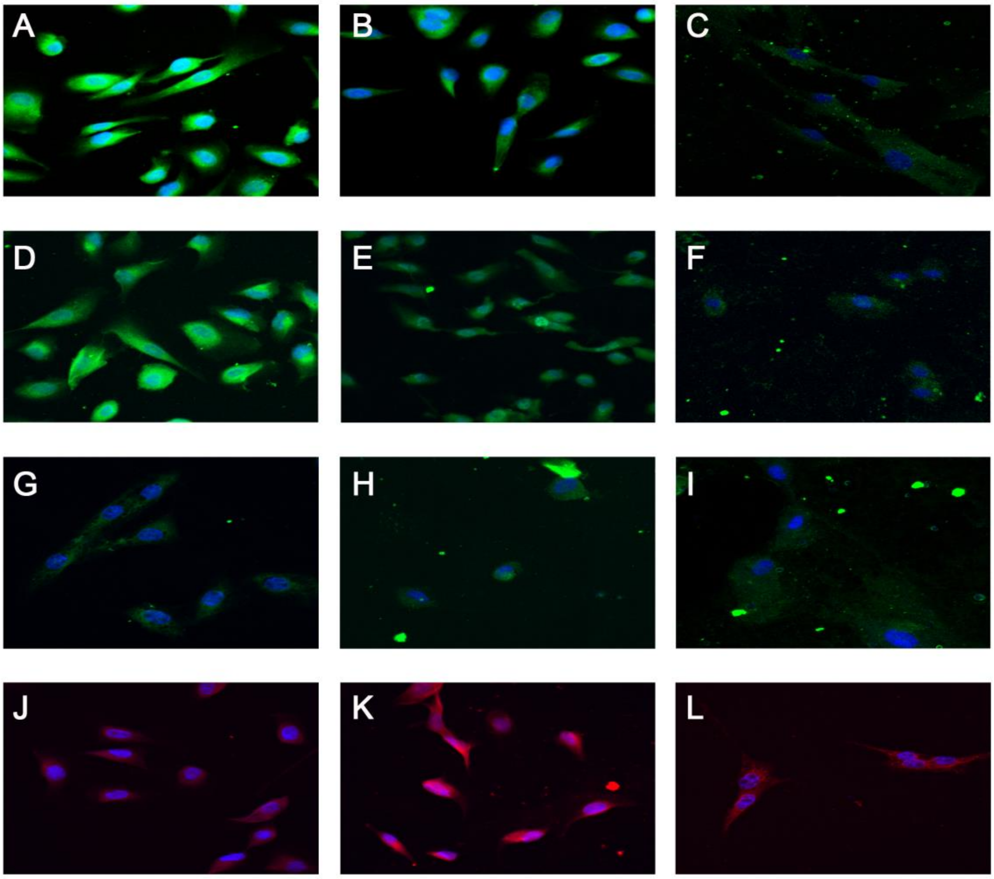

Supplement: Supplementary Information [file srep11660-s1.pdf]
